# Differentiation Between Radiation Necrosis and True Tumour Progression After Radiotherapy to Intracranial Metastases

**DOI:** 10.1111/1754-9485.13847

**Published:** 2025-03-09

**Authors:** Arian Lasocki, Joseph Sia, Stephen L. Stuckey

**Affiliations:** ^1^ Department of Cancer Imaging Peter MacCallum Cancer Centre Melbourne Victoria Australia; ^2^ Sir Peter MacCallum Department of Oncology The University of Melbourne Parkville Victoria Australia; ^3^ Department of Radiology The University of Melbourne Parkville Victoria Australia; ^4^ Department of Radiation Oncology Peter MacCallum Cancer Centre Melbourne Victoria Australia; ^5^ School of Clinical Sciences at Monash Health Monash University Clayton Victoria Australia; ^6^ Department of Medical Imaging and Radiation Sciences Monash University Clayton Victoria Australia

**Keywords:** intracranial metastases, magnetic resonance imaging, radiation necrosis, stereotactic radiosurgery

## Abstract

Differentiating between radiation necrosis and true tumour progression after radiotherapy is challenging due to overlapping imaging appearances. This review outlines useful techniques and imaging features for making this distinction, as well as potential pitfalls. Both radiation necrosis and true tumour progression commonly manifest as peripherally enhancing lesions on post‐contrast T1‐weighted imaging, but the enhancing rim should be thin in radiation necrosis, while more discrete nodular enhancement suggests active tumour. Other features on post‐contrast MRI that suggest radiation necrosis include enhancing lesions across anatomical boundaries, clustering of enhancing lesions and a change in lesion shape. Central diffusion restriction corresponding to the central necrotic area favours radiation necrosis, but there are potential pitfalls to be aware of, including hypercellular tumours, coagulative necrosis due to bevacizumab and intra‐lesional haemorrhage. Radiation necrosis typically results in small, clustered foci of magnetic susceptibility on susceptibility‐sensitive sequences, and the absence of such foci should raise concern for active tumour. When uncertainty remains, ancillary techniques such as MR perfusion and amino acid PET can improve confidence. Atypical appearances of radiation necrosis can occur, for example, cystic radiation necrosis or radiation necrosis occurring after radiotherapy to adjacent structures. It is also important for the radiologist to be aware of additional factors that may increase the likelihood of either radiation necrosis or tumour necrosis or influence patient management.

**Abbreviations**
ASL: arterial spin labelling
DCE: dynamic contrast‐enhanced
DSC: dynamic susceptibility contrast
DWI: diffusion‐weighted imaging
FDG: fluorine‐18‐fluorodeoxyglucose
FET: fluorine‐18‐fluoroethyl‐L‐tyrosine
IM: intracranial metastases
K^trans^
: volume transfer constant
MRS: MR spectroscopy
PET: positron‐emission tomography
rCBV: relative cerebral blood volume
RN: radiation necrosis
RT: radiotherapy
SRS: stereotactic radiosurgery
SWI: susceptibility‐weighted imaging
T1WI: T1‐weighted imaging
TBRmax: maximum tumour‐to‐brain ratio
TBRmean: mean tumour‐to‐brain ratio
TTP: true tumour progression

## 1 Introduction

A diagnosis of intracranial metastases (IM) had traditionally conveyed a poor prognosis, but survival rates have greatly increased recently due to advancements in systemic therapies and radiotherapy techniques. Not all systemic therapies are active intracranially, however, and even for agents that do have some intracranial efficacy, it is not uncommon to observe progression of IM despite an extracranial response. This increases the importance of IM in a patient's disease course, in turn increasing the role of stereotactic radiosurgery (SRS), a highly‐targeted radiotherapy (RT) technique, for the treatment of IM. SRS may be used up‐front in combination with treatments with limited intracranial efficacy, later in the patient's disease course upon evidence of intracranial progression or, increasingly, in combination with systemic treatments that are active intracranially in order to improve response rates [1]. SRS is also often utilised after neurosurgical resection of IM [2], both for management of macroscopic residual or recurrent disease and to eliminate microscopic disease to decrease the likelihood of recurrence. Additionally, there is growing interest in performing SRS pre‐operatively, aiming to reduce the risk of regional nodular leptomeningeal disease due to tumour spillage during resection [3]. SRS is traditionally given as a single fraction, though there is increasing interest in hypofractionated SRS (3–5 fractions) for larger lesions to lower the risk of radiation necrosis without compromising tumour control [4, 5].

SRS can, however, lead to radiation‐induced brain injury, known as radiation necrosis (RN). This is thought to involve vascular changes secondary to endothelial damage [6, 7]. RN has been reported to occur after up to 24% of SRS treatments for IM [4, 8, 9]. It usually occurs 3–18 months after SRS [10], though it can occur even several years later [11]. While the appearances of RN often mimic those of true tumour progression (TTP), we suggest that the term ‘pseudoprogression’ be avoided in the setting of RT to IM. Pseudoprogression in the context of brain tumours typically refers to a more acute, transient local tissue reaction developing within the first 2–6 months after commencing RT for an intracranial glioma, most commonly when used in conjunction with temozolomide chemotherapy [12, 13].

Both RN and TTP typically manifest as new or enlarging contrast‐enhancing lesions a variable period after RT, and this overlap in the imaging appearances provides challenges for accurate diagnosis. Often, RN will regress or stabilise without any specific treatment. Before such regression or plateauing occurs, however, the lesion may enlarge for many months [14], increasing concern and the potential for an adverse outcome if misdiagnosed. Accurate diagnosis is important for optimal patient management. Misdiagnosing RN as TTP can result in unnecessary intervention, including the morbidity of surgical resection of a lesion that may have regressed without treatment, the potential toxicity of a new systemic therapy instigated for presumed tumour progression, cessation of an effective systemic therapy due to an erroneous belief that it is not working, or worsening RN if SRS is repeated. Similarly, a delayed diagnosis of TTP will inevitably lead to progression of active tumour, requiring a larger resection with an increased risk of residual tumour and post‐operative deficits, or the possibility that resection is no longer feasible.

Despite the overlap in imaging appearances, it is possible to provide a likely diagnosis in many cases using the techniques and imaging features described below. The findings vary from lesion to lesion, and they vary in their sensitivity and specificity; thus, a combination of imaging sequences and features should be utilised. A definitive diagnosis may not always be possible; however, suggesting an appropriate management plan is another important role of the radiologist. In many cases, close imaging follow‐up until a more definitive diagnosis can be made will be appropriate. Additionally, cases of possible RN are often challenging from both a diagnostic and management perspective, making multi‐disciplinary input valuable. It is also important to note that much of the published literature on RN focuses on intracranial gliomas rather than IM [15, 16]. While some concepts and imaging features are relevant to both types of tumours, differences in the underlying biology of these tumours have implications for imaging interpretation. This review describes and illustrates imaging techniques and features useful for distinguishing between RN and TTP after SRS to IM, as well as atypical appearances, potential pitfalls and important clinical factors to consider.

## 2 Imaging Techniques

### 2.1 Post‐Contrast T1‐Weighting Imaging

**FIGURE 1:**
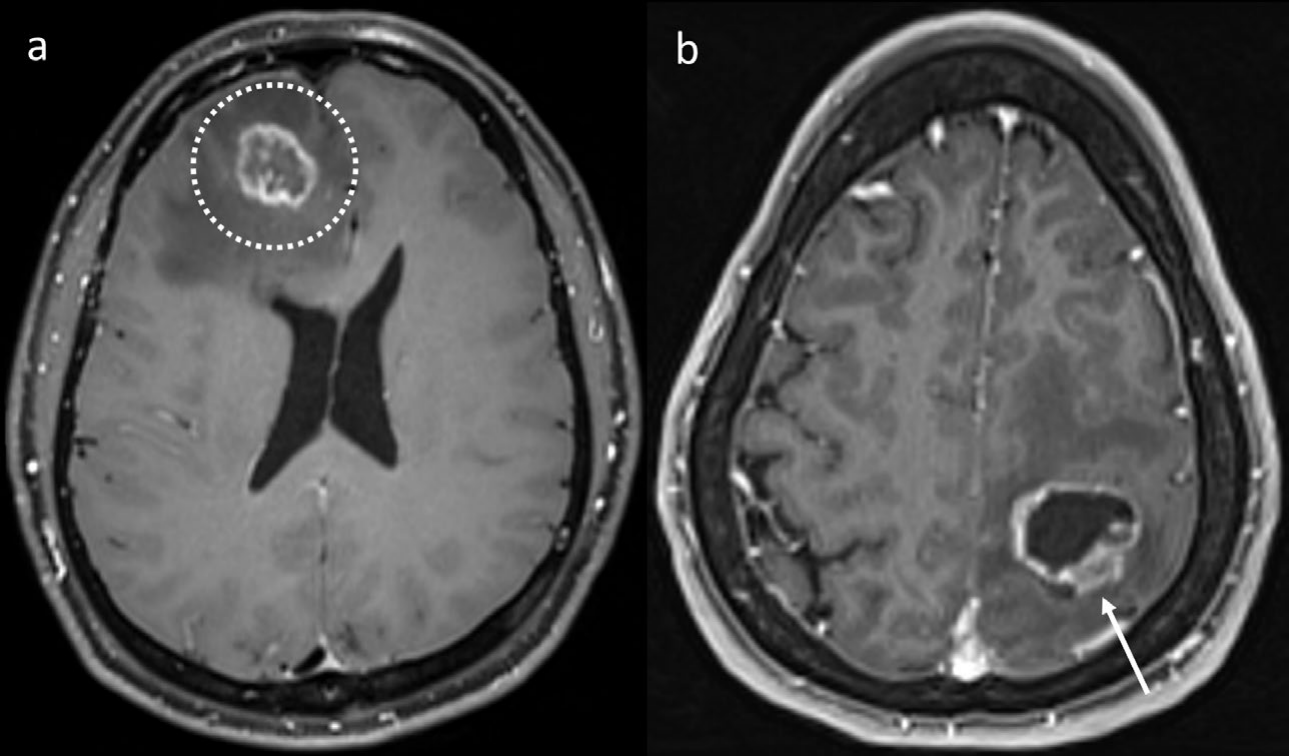
Post‐contrast T1WI demonstrating characteristic appearances of RN (a, dotted circle) and TTP (b). RN typically exhibits thin peripheral enhancement, while the presence of nodular enhancement (arrow) suggests TTP.

**FIGURE 2:**
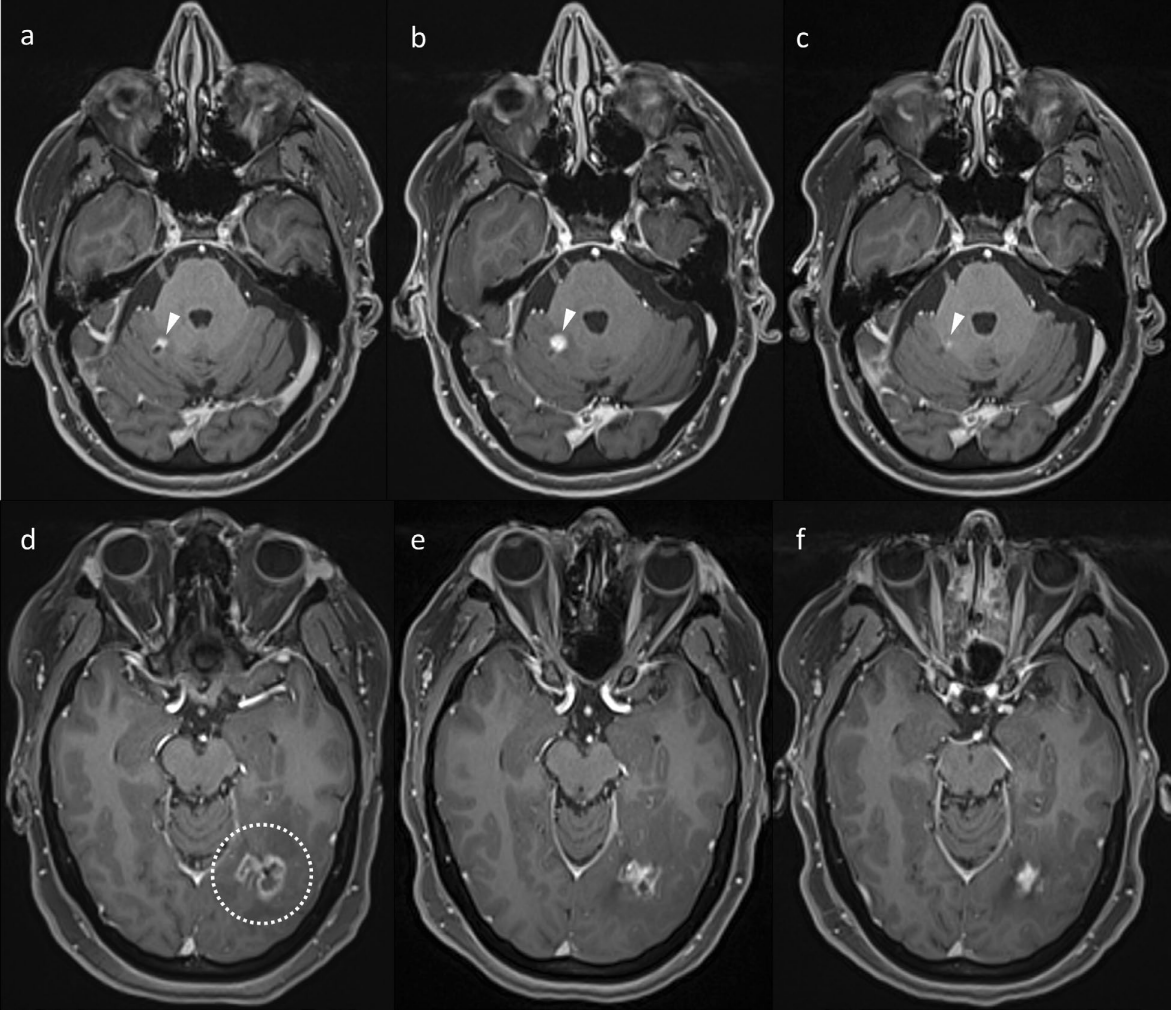
Post‐contrast T1WI demonstrating two examples of when RN may occasionally have more solid appearances. The top row (a–c) shows a small enhancing lesion (arrowhead) which initially enlarged (b), before regressing spontaneously (c), consistent with RN. Due to its small size, the lesion did not develop the central necrosis usually seen in RN. The bottom row (d–f) shows an area of regressing RN (dotted circle), which became more solid in appearance as it contracted (f).

**FIGURE 3:**
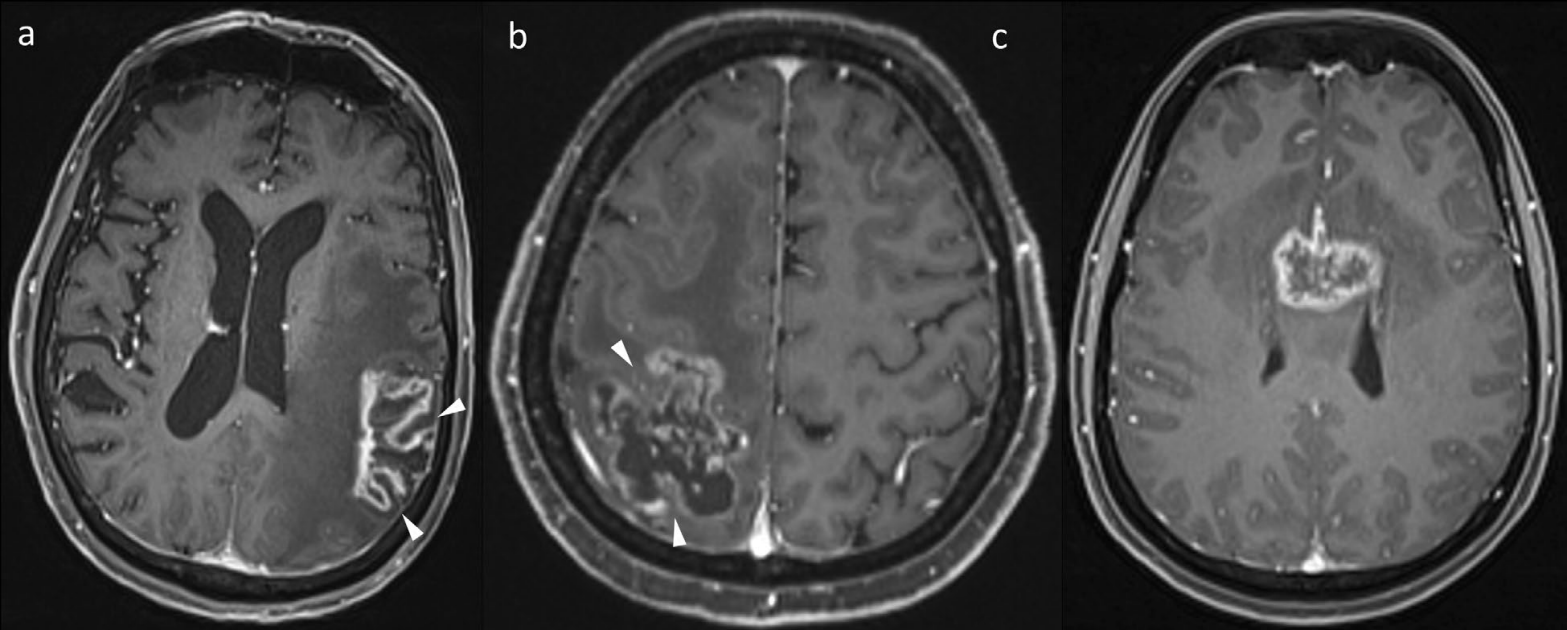
Post‐contrast T1WI showing three examples of different appearances of RN. The lesion in (a) has a wavy lateral border, related to sparing of the cortex (arrowheads). In contrast, the medial border of this lesion is straighter, as it is further from the cortex. (b) shows a classic ‘cut pepper’ appearance, with wavy margins due to sparing of the cortex (arrowheads) and small foci of enhancement within it akin to the ‘seeds’. The callosal lesion in (c) is more round, being further from the cortex, and also has enhancing “seeds” centrally.

Both RN and TTP often manifest as peripherally enhancing lesions with central necrosis on post‐contrast T1‐weighted imaging (T1WI), accounting for the overlap in appearances. With RN, the enhancement is typically thin, albeit irregular, without a discrete solid component (Figure 1). In contrast, thicker or more solid enhancement suggests TTP (see Figure 1), though RN may appear more solid on post‐contrast imaging when lesions are small or regressing (Figure 2). The term “cut pepper” is sometimes used to describe the post‐contrast appearances of RN, though the descriptions and explanations for this appearance in the literature are limited [17]. We attribute the cut pepper appearance to the propensity of RN to affect the white matter [18] and relatively spare the grey matter. This results in a wavy or gyriform margin of lesions located close to the cortex, conforming to the juxtacortical white matter. In contrast, lesions or parts of lesions located away from the cortex are typically more rounded, albeit slightly irregular (Figure 3). It is not the resemblance to a cut pepper that is important, but rather the preferential white matter involvement. Additionally, it is common to observe small foci of enhancement within the centre of an RN lesion, analogous to the ‘seeds’ of a cut pepper (see Figure 3).

**FIGURE 4:**
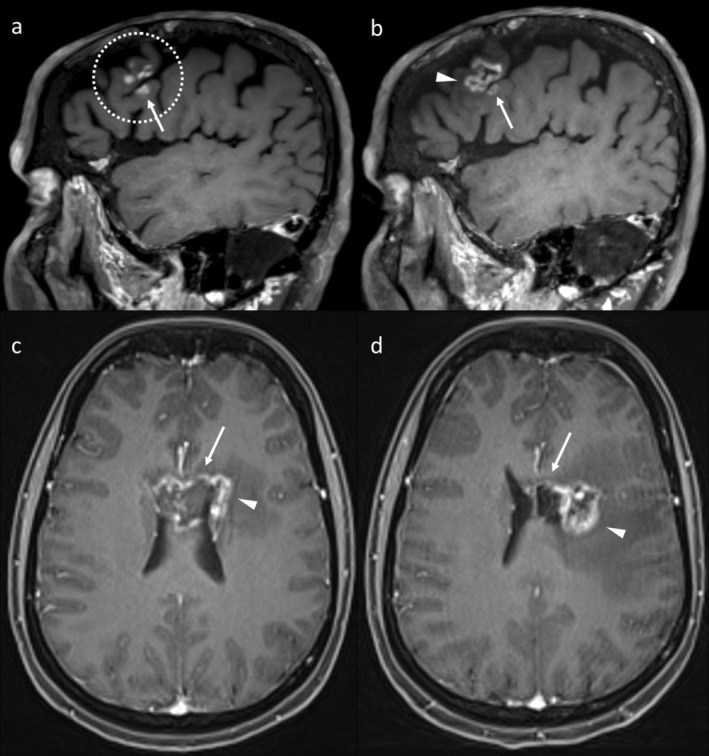
Serial post‐contrast T1WI of two examples of RN demonstrating how a more nuanced interpretation of conventional images can improve diagnostic confidence. In the first example (top row), several small enhancing lesions developed within the RT field (a, dotted circle). Of specific note, one of the lesions (arrow) developed on the opposite side of a sulcus (arrow). The lesions evolved differently (b), with the superior cluster coalescing (arrowhead) and the separate smaller lesion (arrow) remaining stable. The second example (c–d) demonstrates a change in the shape of the lesion over time, with regression of a callosal component (arrow) occurring simultaneously with the enlargement of a caudate component (arrowhead).

While RN and TTP can have similar appearances on conventional sequences, a more nuanced interpretative approach can increase their value [19]. One feature favouring RN is the development of enhancing lesion(s) across an anatomical boundary from the initial IM, as this appearance would be less typical of IM [19]. Such boundaries include dural reflections, sulci and the ventricles. A second useful feature is clustered enhancing lesions within the RT field. Individual lesions may evolve differently and may coalesce over time. A third useful feature is a change in lesion shape [19]. As RN is a dynamic process, different parts of the lesion may be at different stages of their evolution, potentially manifesting as enlargement of one part of the lesion at the same time as another component regresses, producing a change in the shape of the lesion [19]. Examples of these features are provided in Figure 4. Knowledge of the RT volumes is helpful when interpreting these appearances, though even when they are not available, the parts of the brain at risk of RN can be estimated by visualising a margin around the original lesion on imaging performed for treatment planning.

### 2.2 Diffusion‐Weighted Imaging

**FIGURE 5:**
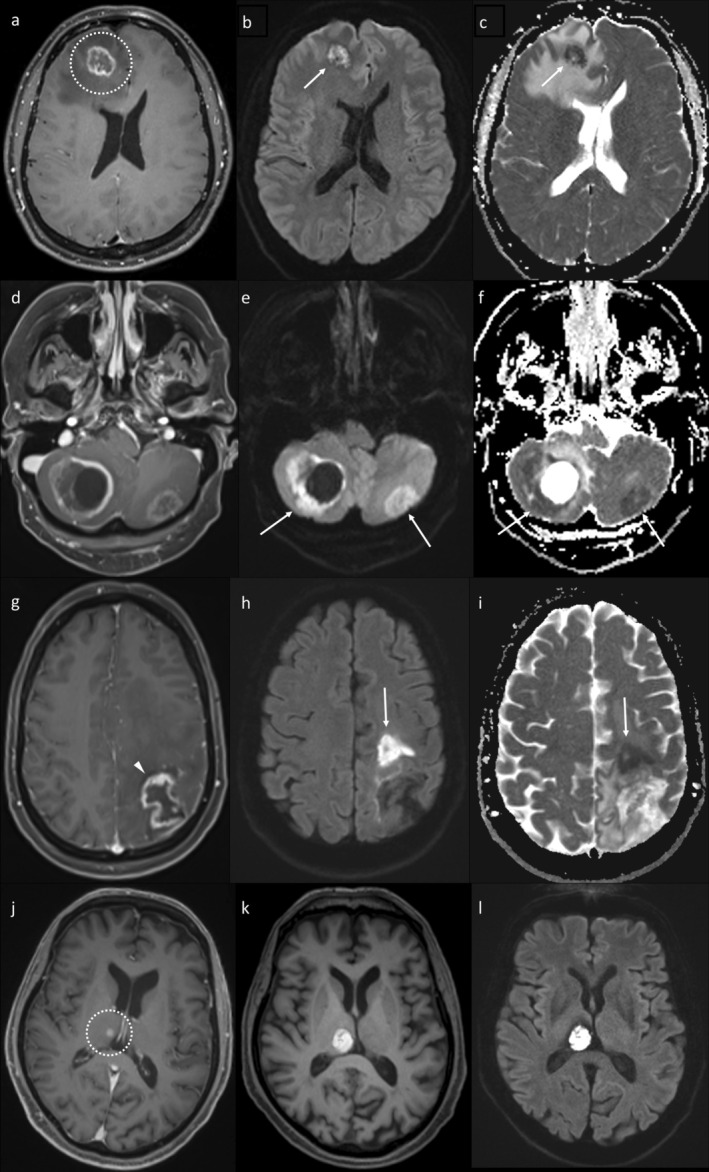
Different examples of the value of DWI in diagnosing RN and potential pitfalls. The top row (a–c) is an example of RN (dotted circle) demonstrating diffusion restriction (arrows) in the central non‐enhancing portion, with high signal on DWI (b) and low signal on the ADC map (c). The second row (d–f) demonstrates diffusion restriction in two cerebellar lesions in a patient with small cell lung cancer. Importantly, while there is some central diffusion restriction (high DWI signal, e, and low ADC signal, f), especially in the left cerebellar lesion, there is also diffusion restriction along the enhancing periphery; true tumour progression was confirmed at surgical resection of both lesions. The third row (g–i) shows an example of RN on post‐contrast T1WI (g) treated with bevacizumab, resulting in an area of presumed coagulative necrosis on subsequent imaging (presumably occurring within an area of less severe radiation injury), with high DWI signal (h) and low ADC (i). Other than for a decrease in the enhancement related to bevacizumab, the original lesion remained stable, confirming RN. The fourth row (j–l) demonstrates a metastasis (j, dotted circle), which developed haemorrhage, shown as high signal on pre‐contrast T1WI (k) and DWI (l).

Advanced MRI sequences are important for improving diagnostic confidence. A helpful feature predicting RN on diffusion‐weighted imaging (DWI) is central diffusion restriction [20], somewhat similar in appearance to that occurring with cerebral abscess [21, 22]. This was initially described as a predictor of RN in intracranial gliomas [23, 24] and has subsequently been shown to also have value in the context of IM [20]. There are some mimics to consider, however. Firstly, hypercellular metastases from primaries such as small cell lung cancer can produce diffusion restriction in areas without distinct solid enhancement. Despite a lack of enhancement, such areas can appear more solid than areas of necrosis, for example, on T2‐weighted imaging. Additionally, the enhancing periphery will often diffusion‐restrict in addition to the centre, whereas diffusion restriction should only be considered suggestive of RN if confined to the necrotic centre [20]. Coagulative necrosis after treatment with bevacizumab can also cause diffusion restriction, usually at sites that have received substantial RT doses [25], though the diagnosis will generally be clear based on a temporal relationship with commencing bevacizumab therapy. Evidence of coagulative necrosis in this context would not be expected to reflect underlying active tumour. Additionally, intra‐lesional haemorrhage can mimic diffusion restriction. Examples of these different appearances on DWI, including potential mimics, are provided in Figure 5.

### 2.3 Susceptibility‐Weighted Imaging

A susceptibility‐sensitive sequence such as susceptibility‐weighted imaging (SWI) also provides valuable information. RN is typically associated with the development of multiple small foci of magnetic susceptibility, often increasing in number over time. These foci can occur within the enhancing periphery, within the necrotic centre, in the adjacent brain, or in a combination of these sites. This appearance is akin to the scattered foci of magnetic susceptibility that may be seen after whole‐brain RT or craniospinal irradiation [26], but more concentrated and occurring earlier after RT. While we have found this appearance supportive of a diagnosis of suspected RN, TTP can itself result in magnetic susceptibility unrelated to SRS due to intra‐tumoural haemorrhage; thus, this feature is not specific for RN. More importantly, perhaps, it is uncommon in our experience to observe RN in the absence of such foci of magnetic susceptibility; thus, their absence should raise concern for TTP. Larger haemorrhages can occur both in TTP and after RT itself, and thus provide limited discriminative value.

### 2.4 MR Perfusion

**FIGURE 6:**
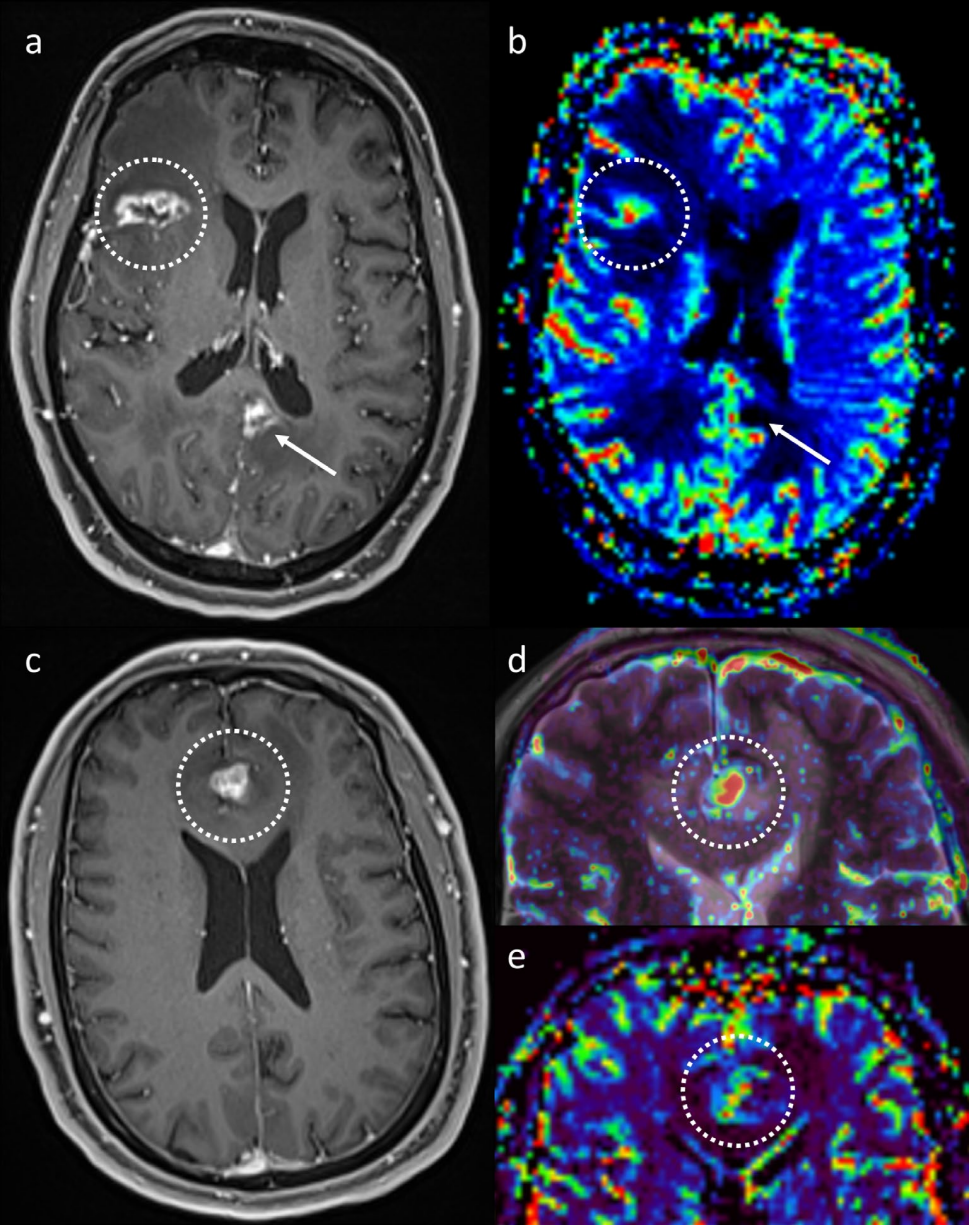
The first patient (top row) has two peripherally enhancing lesions on post‐contrast T1WI (a) that had enlarged after earlier treatment with SRS. The lesion involving the right frontal operculum and insula (dotted circle) demonstrates high rCBV and was confirmed to represent TTP at resection. In contrast, the left posterior cingulate lesion did not demonstrate rCBV elevation and was confirmed as RN based on subsequent regression. The second patient (bottom row) has an enhancing lesion (c, dotted circle), which demonstrated elevated K^trans^ on DCE perfusion (d) and was confirmed to represent tumour at resection. The rCBV elevation on DSC perfusion (e) was less marked, highlighting the importance of using a combination of sequences and features.

MR perfusion techniques are also an important adjunct (Figure 6), though there is less literature on their use in intracranial metastases than in gliomas [15, 16]. Dynamic susceptibility contrast (DSC) perfusion is the most established perfusion technique in this context, and indeed in brain tumour imaging more broadly. TTP is associated with higher relative cerebral blood volume (rCBV) than RN, though the values and suggested thresholds vary across studies [16, 27, 28, 29]. In practice, visual assessment is adequate [30]. One limitation with DSC perfusion, however, is that it is affected by blood products, which are commonly present in intracranial metastases [31, 32], and this can result in false negative DSC perfusion results. Dynamic contrast‐enhanced (DCE) perfusion has theoretical benefits in such a setting, being less affected by blood products, though there is less literature on its use [33, 34]. DCE perfusion provides a different set of output parameters, such as the volume transfer constant, K^trans^ [33]. Arterial spin labelling (ASL), which is performed without IV contrast, has also been studied less extensively than DSC perfusion [34] and patients will generally already be receiving gadolinium in this setting. Additionally, due to the inherently lower signal of ASL [35], even active metastases may produce little ASL signal [36].

Ultimately, however, perfusion is just a tool like all the other MRI techniques discussed earlier, and both false positives and false negatives can occur [14]. In our experience, perfusion may be suspected to falsely suggest TTP if the appearances are otherwise most consistent with RN, though earlier follow‐up may be prudent. We find perfusion to be most helpful in equivocal cases or to provide support when TTP is suspected. At our institution, we consider DSC and DCE perfusion to be complementary and routinely perform both in this context, with the contrast used for DCE perfusion providing the pre‐load for the DSC perfusion, though overall we find DSC perfusion to be the more helpful of the two.

### 2.5 MR Spectroscopy

Similar to some of the other techniques already described, there is less literature regarding the use of MR spectroscopy (MRS) after SRS to IM than in gliomas. Differences in the underlying biology between gliomas and IM are particularly relevant to MRS, in turn having implications for interpretation. A key feature of intracranial gliomas is the presence of infiltrating tumour cells beyond the contrast‐enhancing area, which has relevance to several advanced MRI techniques, including MRS, perfusion and DWI. With respect to MRS, glioma progression may be suggested over RN by the presence of increased levels of choline adjacent to the enhancing area, as a reflection of increased cell membrane turnover, typically assessed as ratios compared to creatine and N‐acetyl‐aspartate [16]. In contrast, a metastasis is generally assumed to be confined to the enhancing area, accounting for the lower diagnostic utility of MRS in this context [16, 27].

MRS is also more challenging logistically in the context of IM. The lesion(s) of concern need to be specifically selected, but this may not be readily apparent to the MRI technologist or supervising radiologist at the time of the scan. It is common for multiple enhancing lesions to be present in such patients, and determining which lesions could potentially represent RN may require comparison with previous imaging while the patient is being scanned, extending the examination.

### 2.6 Delayed‐Contrast MRI

Delayed‐contrast MRI is a technique, which utilises a second post‐contrast acquisition about 75 min after contrast administration, which is then compared to the initial post‐contrast acquisition to identify areas with contrast wash‐out versus contrast accumulation [37]. Initial research suggested that a lesion with a higher proportion of contrast wash‐out was indicative of active tumour [37]. However, a more recent study has suggested that this may simply reflect a greater proportion of enhancing tissue compared to central necrosis, rather than a true difference in the underlying tissue properties [38]. The uptake of this technique in routine clinical practice seems limited, and patient and logistic inconvenience (given the need for a repeat acquisition 75 min later) is also a disadvantage.

### 2.7 Amino Acid Positron‐Emission Tomography

Despite the range of MRI tools at the radiologist's disposal, the diagnosis may remain uncertain. One technique gaining popularity is positron‐emission tomography (PET) using amino acid tracers such as FET (fluorine‐18‐fluoroethyl‐L‐tyrosine). The advantage over the most commonly used PET tracer, FDG (fluorine‐18‐fluorodeoxyglucose), is a lack of tracer uptake in the normal brain [39]. Both the tracer uptake curve and tumour‐to‐brain ratio should be assessed. A pattern I curve (constantly increasing uptake) favours RN, while a pattern II curve (early peak then plateau) or pattern III curve (early peak then descent) favour TTP [40]. A maximum tumour‐to‐brain ratio (TBRmax) > 2.55 and mean tumour‐to‐brain ratio (TBRmean) > 1.95 also predict TTP [40]. The more objective nature of amino acid PET is likely to be most valuable in cases that are equivocal on MRI or when the reporting radiologist is less comfortable distinguishing between RN and TTP with MRI. However, our experience suggests that the accuracy of amino acid PET is lower when the appearances on comprehensive MRI assessment are truly equivocal after subspecialist review. As such, we generally reserve its use for patients for whom short‐term MRI follow‐up is considered risky, for example, when further lesion enlargement might compromise the ability to achieve a satisfactory surgical resection.

## 3 Dual Pathology

**FIGURE 7:**
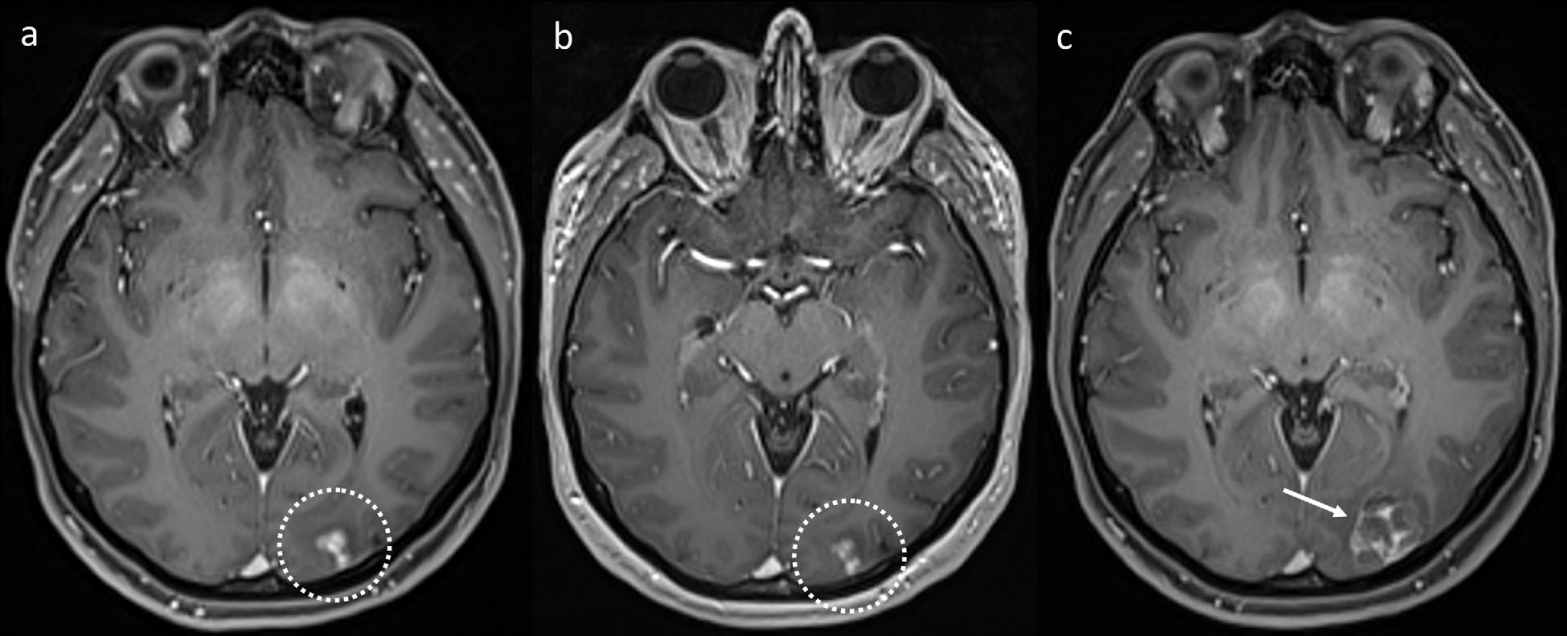
Axial T1WI over time in a patient who developed enhancement at the site of previous SRS (a, dotted circle). This improved without treatment (b), but later a nodular enhancing lesion developed at the same site (c, arrow), confirmed to represent TTP at resection.

When RN occurs in the case of glioblastomas treated with RT, there will usually still be some active tumour. A single process is most common for IM treated with SRS, but RN and TTP can occasionally coexist. In such cases, two morphologically distinct components of the lesion may be observed, for example, a more nodular enhancing area representative of the tumour, with a peripherally enhancing area either surrounding or to one side of the nodular area, representative of concomitant RN. Additionally, being a dynamic process, TTP may manifest after changes of RN have regressed (Figure 7). RN and TTP can also occur in different lesions in the same patient (see Figure 6, top row), either at the same time point or across the patient's course. Therefore, it is important to keep an open mind and critically appraise each individual lesion on each study. Furthermore, there remains an ongoing possibility of new IM developing; thus, ongoing MRI follow‐up remains important even if RN has been diagnosed definitively.

## 4 Atypical Appearances

Most cases of RN manifest as lesions with irregular peripheral enhancement, but cystic or predominantly cystic RN can occasionally occur. Additionally, while we have focused on RN after treatment of IM, it can also occur after SRS to benign lesions, such as meningiomata. Another atypical manifestation of RN is that occurring after RT to lesions in the head and neck, calvarium or scalp, which can be mistaken for metastases. There is often a longer interval between RT and the development of new enhancing lesions in such cases, and it is common for the appearances to persist for longer. Lesions usually affect the white matter, as for RN occurring after SRS to IM, and are often multiple and/or ill‐defined. It is important that a history of RT to adjacent structures is provided by the referring clinician, even if this was not targeted to the brain, as misdiagnosis is likely without this history.

## 5 Clinical Considerations

SRS is associated with high rates of local control of over 80% [2], thus TTP is uncommon at sites of previous SRS. In contrast, RN may occur in up to 24% of SRS treatments of IM [4, 8, 9]. While TTP can appear similar to RN, in our experience many cases of TTP can be confidently diagnosed as such using standard MRI sequences, for example, based on the presence of distinct nodular enhancement. As a result, for cases in which the lesion has appearances potentially compatible with RN and there is genuine uncertainty regarding the diagnosis, RN is statistically more likely than TTP, which has implications for interpretation. Our practice is to generally favour a conservative approach when RN is a possibility and there are no features that would more specifically suggest TTP, following up with MRIs at appropriate intervals and awaiting more definitive imaging evidence of one of the two entities. We generally use an MRI follow‐up interval of 6–12 weeks, depending on a range of factors, such as the rate of enlargement, location and treatment options.

It is important for the reporting radiologist to be aware of clinical factors which may alter the likelihood of either RN or TTP, and hence for this information to be provided by the referring clinician. For example, the risk of RN will increase with the RT dose, especially if RT has been administered more than once. This could include SRS on a background of previous whole‐brain RT, SRS after previous RT to adjacent structures (such as the scalp) with some dose administered to the brain, or repeat SRS. The RT technique used is also relevant. For brain tumours, RT techniques can generally be categorised as stereotactic or non‐stereotactic. Stereotactic RT techniques provide a sharp radiation dose fall‐off around the target volume, thus resulting in minimal collateral irradiation of neighbouring normal tissue. Stereotactic RT can be delivered using various RT machines such as a linear accelerator (LINAC), CyberKnife (a LINAC on a robotic arm), or GammaKnife (an RT machine housing 192 Cobalt‐60 radionuclides specifically designed for intracranial SRS). The gradient of dose fall‐off can further vary between these SRS techniques, being very sharp with the GammaKnife and less so with the LINAC. Non‐stereotactic techniques are typically delivered using a conventional LINAC and give a slower dose fall‐off outside the target volume, thus RN can occur across a wider area.

A knowledge of the patient's prior response to SRS may also be relevant. For example, tumour histology, including tumour subtypes based on additional factors such as mutation status in lung cancer and hormonal status in breast cancer, influences response to RT [41]. As such, a history of previous TTP may suggest a tumour phenotype that is less responsive to radiotherapy. Similarly, patients vary in their degree of radiosensitivity due to genetic differences [42], thus a history of prior RN may suggest a greater susceptibility to RN. It is also worthwhile being aware of any concomitant or previous treatment with immunotherapy, though the literature on the link between immunotherapy and a higher risk of RN is heterogeneous. Concurrent SRS and immunotherapy are being increasingly utilised and deliver improved outcomes [43]. Fortunately, despite some research suggesting an increased risk of RN when SRS is combined with immunotherapy [44], more recent meta‐analyses conclude that the risk is not significantly increased [43, 45]. We suggest that imaging interpretation should be the same even if a patient is being treated with immunotherapy.

RN is often asymptomatic, but symptoms can occur, often related to the surrounding oedema. Occasionally, surgical resection may be warranted for symptom control and mass effect despite a suspected diagnosis of RN on imaging, and it is important for the radiologist to recommend a neurosurgical opinion in such cases. This is predominantly a consideration if the patient's metastatic disease is otherwise well controlled. It is also important to consider other factors related to the lesion and the patient when determining appropriate management, such as the management implications if the lesion enlarges further. For example, conservative management of an equivocal lesion may be more appealing if the lesion is located further from eloquent brain, as further enlargement may have little impact on the ability to achieve a satisfactory resection without additional morbidity. In contrast, there may be a lower threshold for considering resection if further lesional enlargement would affect eloquent brain.

## 6 Treatment of Radiation Necrosis

In the past, the main non‐invasive option for the management of RN was corticosteroids, but this is associated with a range of side effects, including decreasing the efficacy of immunotherapy. This has led to the growing use of bevacizumab, a monoclonal antibody against vascular endothelial growth factor, for symptomatic management [7]. Bevacizumab also decreases lesional enhancement, making it difficult to assess the lesion itself. Bevacizumab can sometimes be used when the diagnosis of RN is equivocal, although this should generally be reserved for when surgery is relatively contraindicated (e.g., due to a challenging lesion location or medical co‐morbidities) due to the bevacizumab wash‐out period prior to surgery. If the history of bevacizumab therapy is not known or not appreciated, improved imaging appearances may be erroneously interpreted as evidence of RN. Another imaging feature associated with bevacizumab therapy is a new area(s) of diffusion restriction due to coagulative necrosis [25], as noted above (see Figure 5).

## 7 Conclusion

The distinction between RN and TTP can be challenging in the setting of imaging after SRS to IM. It is important for the radiologist to consider the full variety of imaging features and techniques in order to provide the most likely diagnosis and inform subsequent patient management. Despite the overlap in the appearances, routine post‐contrast T1WI is the mainstay of the assessment, and with clinician experience and a more nuanced interpretation, a confident distinction between RN and TTP is possible in many cases. DWI and SWI also provide important information and should be performed routinely in patients with IM. When uncertainty remains, ancillary techniques such as MRI perfusion and amino acid PET can improve confidence. Generous use of follow‐up imaging is important to provide further diagnostic validation or potentially suggest revision of the suspected diagnosis and for the ongoing assessment of a response to treatment.

## Ethics Statement

Institutional ethics committee approval was received.

## Consent

Ethics approval included approval for a waiver of patient consent.

## Conflicts of Interest

The authors declare no conflicts of interest.

## Data Availability

The authors have nothing to report.
